# Early postoperative weight gain is associated with increased risk of graft failure in living donor liver transplant recipients

**DOI:** 10.1038/s41598-019-56543-3

**Published:** 2019-12-27

**Authors:** Hye-Won Jeong, Kyeo-Woon Jung, Seon-Ok Kim, Hye-Mee Kwon, Young-Jin Moon, In-Gu Jun, Jun-Gol Song, Gyu-Sam Hwang

**Affiliations:** 1 Department of Anaesthesiology and Pain Medicine, International St. Mary’s Hospital, Catholic Kwandong University College of Medicine, Incheon, Korea; 2 Department of Anaesthesiology and Pain Medicine, Laboratory for Cardiovascular Dynamics, Asan Medical Centre, University of Ulsan College of Medicine, Seoul, Korea; 3 Department of Clinical Epidemiology and Biostatistics, Asan Medical Centre, University of Ulsan College of Medicine, Seoul, Korea

**Keywords:** Hepatology, Outcomes research

## Abstract

Fluid overload (FO) has been shown to adversely affect multiple organs and survival in critically ill patients. Liver transplantation (LT) carries the risk of massive transfusion, which frequently results in FO. We investigated the association of postoperative weight gain with graft failure, early allograft dysfunction (EAD), and overall mortality in LT. 1833 living donor LT (LDLT) recipients were retrospectively analysed. Patients were divided into 2 groups according to postoperative weight gain (<3% group [n = 1391] and ≥3% group [n = 442]) by using maximally selected log-rank statistics for graft failure. Multivariate Cox and logistic regression analyses were performed. The ≥3% group was associated with graft failure (adjusted HR [aHR], 1.763; 95% CI, 1.248–2.490; *P* = 0.001). When postoperative weight change was used as a continuous variable, the aHR for each 1% increase in postoperative weight was 1.045 (95% CI, 1.009–1.082; *P* = 0.015). In addition, the ≥3% group was associated with EAD (adjusted OR [aOR], 1.553; 95% CI, 1.024–2.356; *P* = 0.038) and overall mortality (aHR, 1.731; 95% CI, 1.182–2.535; *P* = 0.005). In conclusion, postoperative weight gain may be independently associated with increased risk of graft failure, EAD, and mortality in LDLT recipients.

## Introduction

Liver transplantation (LT) carries the risk of massive transfusion, which frequently results in fluid overload (FO). There is growing evidence that FO can be harmful to critically ill patients or those undergoing major abdominal surgery^1–6^, with the adverse effects persisting even after the fluid status has improved^7^. LT recipients may be particularly vulnerable to FO owing to their fragile cardiopulmonary status, resulting in increased complications and prolonged stay in the intensive care unit (ICU)^8–10^.

Graft survival after LT has been found to be associated with various factors, including donor and recipient age, model for end-stage liver disease (MELD) score, and ischaemic time^11,12^. Meanwhile, FO has been reported to have adverse impacts on multiple organ systems^13,14^. FO may cause endothelial dysfunction and bacterial or endotoxin translocation, leading to a state of systemic inflammation^15,16^, which may result in decompensation and multiorgan failure in advanced liver cirrhosis patients^17^. Systemic inflammation may also provoke hepatic angiogenesis of liver transplants, leading to fibrosis and cirrhotic remodelling that ultimately result in decreased graft survival in LT recipients^18–20^.

Despite the link between FO, inflammation, and organ or graft dysfunction, the impact of perioperative FO on graft function in LT recipients has not been well elucidated. Therefore, we investigated the relationship between early postoperative weight gain and the occurrence of graft failure in living donor LT (LDLT) recipients. We also evaluated the occurrence of early allograft dysfunction (EAD), as well as overall mortality.

## Methods

This retrospective observational study was approved by the Institutional Review Board of Asan Medical Centre (Seoul, South Korea), which did not require written informed consent due to the retrospective design of the study. All methods were performed in accordance with the relevant guidelines and regulations.

### Patients

All patients who underwent LT at our institution, from January 2008 to September 2015, were enrolled. The Hospital Based Organ Procurement Organization designated by law procured living or deceased liver grafts, and none of the grafts were obtained from executed prisoners. We excluded patients (1) who were <18 years old, (2) who underwent orthotopic LT, and (3) whose preoperative serum creatinine level was >1.5 mg/dL. The clinical data of the remaining patients, including demographic data, liver disease, perioperative laboratory data, donor data, intraoperative data containing anaesthetic management and surgical data, and postoperative morbidity and mortality were obtained from the computerized databases (electronic medical records system) of our institution, and analysed.

### Intraoperative management

Anaesthesia and haemodynamic monitoring were performed according to our institutional standard^21^. Briefly, we maintained anaesthesia with sevoflurane or desflurane, a mixture of 50% O_2_ and 50% air, and a continuous infusion of fentanyl. Arterial pressure was monitored using radial and femoral arterial catheters. For advanced haemodynamic monitoring, a pulmonary arterial catheter was inserted and connected to a Vigilance device (Vigilance II®, Edwards Lifesciences, Irvine, CA, USA). According to the patient’s blood pressure and haemodynamics, the attending anaesthesiologist administered either fluid or vasoactive drugs. In cases of low systemic vascular resistance, a continuous infusion of norepinephrine or vasopressin was used to maintain the mean arterial blood pressure either above the preoperative level or 65 mmHg. Intraoperative transfusions of packed red blood cells (pRBCs), fresh frozen plasma (FFP), cryoprecipitate, and apheresis platelets were based on institutional standards adopted from clinical and rotational thromboelastometry guidelines, with the aim of maintaining prothrombin time (international normalized ratio [INR]) <2.0, fibrinogen >100 mg/dL, and platelet counts >30,000/μL.

### Postoperative management

In the postoperative period, our institution’s goal of fluid management is to maintain near-zero fluid balance to minimize weight changes in the ICU^22,23^. Because FO may lead to graft congestion, especially in LDLT recipients, LT recipients are weighed every day and their fluid balance is adjusted accordingly. Patients with massive ascites before surgery are managed with a more liberal fluid management strategy to prevent renal dysfunction such as acute kidney injury (AKI).

### Postoperative weight change

Postoperative weight change (%) was calculated using the following formula: ([body weight on the morning after surgery − body weight on the morning before surgery]/body weight on the morning before surgery) × 100.

### Inflammatory parameters

Serum C-reactive protein (CRP) and neutrophil-to-lymphocyte ratio (NLR) were routinely measured every morning from the preoperative day to postoperative day (POD) 7. Serum CRP level was measured using a latex-enhanced immunoturbidimetric assay on the Cobas 8000 analyser (Roche Diagnostics, Mannheim, Germany), with the CRPL3 reagent (Roche). The diagnostic range of this assay is 0.03 to 35 mg/dL, with a reference value of <0.6 mg/dL. NLR is a marker of systemic inflammation that can be easily obtained from the differential white blood cell count. The NLR was obtained by dividing absolute neutrophil counts by absolute lymphocyte counts.

### Outcome variables

The main outcome of this study was the occurrence of graft failure after LDLT. Graft failure was defined as the earlier of re-transplantation or death from any cause. Other outcomes included EAD, overall mortality, and lengths of ICU and hospital stay. EAD was defined by the presence of one or both of the following variables: bilirubin >10 mg/dL or prothrombin time (INR) >1.6 on POD 7^24^. Graft failure and overall mortality were assessed using electronic medical records and a registry that was regularly updated by the Asan Organ Transplantation Centre. In addition, the prevalence of pulmonary complications or AKI after LDLT was assessed. According to the Kidney Disease: Improving Global Outcomes (KDIGO) criteria, AKI was defined as an increase in serum creatinine by ≥0.3 mg/dL within 48 hours or an increase in serum creatinine to ≥1.5 times baseline, which is known or presumed to have occurred within the prior 7 days^25^.

### Statistical analysis

Continuous variables are reported as mean ± standard deviation or median (interquartile range [IQR]), and categorical variables as frequency (percentage). Between-group comparisons were performed using the Student’s t-test or Mann-Whitney U-test for continuous variables, and the chi-square test or Fisher’s exact test for categorical variables, as appropriate.

We used maximally selected log-rank statistics from the Maxstat R package to determine the optimal cut-off value of postoperative weight change (%) predicting graft failure after LDLT^26,27^. This package tests all possible cutpoints to identify the cutpoint where the maximum of the log-rank statistics is achieved. All patients were classified into 2 groups according to the cutpoint that best discriminates the patients for overall graft survival and failure. The association between postoperative weight change and clinical outcomes after LDLT was evaluated using Cox or logistic regression models. Variables with *P*-values < 0.1 in univariate analyses were entered into multivariate analyses. Multivariate Cox regression analysis was used to assess the factors associated with graft failure and overall mortality. Multiple logistic regression analysis was used to identify the factors associated with EAD. A backward elimination process was used to develop the final multivariate model, and adjusted hazard ratios (aHRs) or adjusted odds ratios (aORs) with 95% confidence intervals (CIs) were calculated. The cumulative graft and patient survival rates were calculated using the Kaplan-Meier method, and log-rank tests were used to evaluate the differences between groups.

In addition, the potential nonlinear relationship between postoperative weight change and risk of graft failure was assessed in a multivariate Cox regression model, in which postoperative weight change was modelled with restricted cubic splines with 5 knots (5^th^, 27.5^th^, 50^th^, 72.5^th^, and 95^th^ percentiles, as suggested by Harrell)^28^; fully aHRs with 95% CIs were plotted against postoperative weight change.

All reported *P*-values are two-sided, and *P*-values < 0.05 were considered statistically significant. SPSS 22.0 (SPSS Inc., Chicago, IL, USA) and R 3.5.1 (R Foundation for Statistical Computing, Vienna, Austria) were used for data manipulation and statistical analyses.

## Results

**Figure 1:**
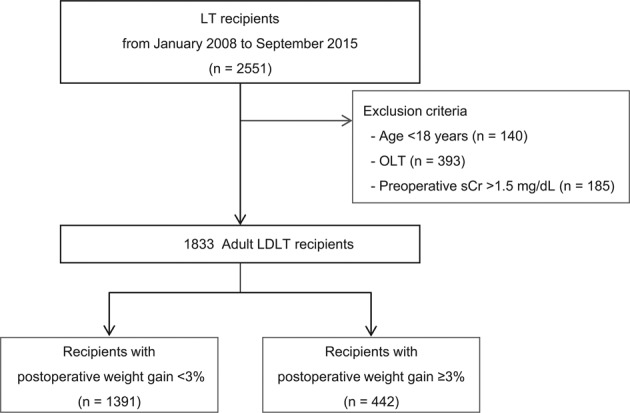
Flowchart of the study population. Abbreviations: LT, liver transplantation; OLT, orthotopic liver transplantation; sCr, serum creatinine; LDLT, living donor liver transplantation.

A total of 1833 adult LDLT recipients were included in the final analysis (Fig. 1), and the median postoperative follow-up time was 5.1 years (IQR, 3.4–7.0). The incidence of graft failure was 7.7% (n = 142). The incidence of EAD was 10.4% (n = 190), and the overall mortality was 6.5% (n = 119). By using the cut-off value determined by the maxstat R package, the 1833 patients were divided into 2 groups based on the amount of postoperative weight gain (<3% group [n = 1391] and ≥3% group [n = 442]).

Table 1 shows the baseline characteristics and intraoperative data of the 2 groups. The postoperative weight gain ≥3% group were older, more likely to be female, and had been more frequently diagnosed with diabetes mellitus or hepatic encephalopathy than the postoperative weight gain <3% group. In addition, the ≥3% group had larger volumes of crystalloid and albumin infusion, higher amounts of pRBC and FFP transfusion, higher urine output, less intraoperative vasopressor use, and longer warm ischaemic and operation time than the <3% group.

**Table 1:** Baseline characteristics and intraoperative data of living donor liver transplantation recipients and donors.

	Total (N = 1833)	Weight gain <3% (n = 1391)	Weight gain ≥3% (n = 442)	*P*-value
**Demographic**				
Age (years)	53.0 (49.0–57.0)	53.0 (48.0–57.0)	54.0 (49.0–58.0)	0.004
Sex, male	1383 (75.5%)	1081 (77.7%)	302 (68.3%)	<0.001
Body mass index (kg/m^2^)	23.8 ± 3.3	24.0 ± 3.2	23.0 ± 3.2	<0.001
Diabetes mellitus	385 (21.0%)	268 (19.3%)	117 (26.5%)	0.002
Hypertension	236 (12.9%)	173 (12.4%)	63 (14.3%)	0.362
Coronary arterial disease	65 (3.5%)	44 (3.2%)	21 (4.8%)	0.154
**Liver disease**				
Fulminant hepatic failure	78 (4.3%)	57 (4.1%)	21 (4.8%)	0.647
Model for end-stage liver disease score	12.0 (9.0–18.0)	12.0 (9.0–18.0)	12.0 (9.0–18.0)	0.951
Hepatic encephalopathy	251 (13.7%)	169 (12.1%)	82 (18.6%)	0.001
Ascites	554 (30.2%)	466 (33.5%)	88 (19.9%)	<0.001
Spontaneous bacterial peritonitis	99 (5.4%)	80 (5.8%)	19 (4.3%)	0.291
Combined hepatocellular carcinoma	927 (50.6%)	685 (49.2%)	242 (54.8%)	0.050
**Laboratory data**				
Haemoglobin (g/dL)	11.0 ± 2.2	11.0 ± 2.2	11.1 ± 2.1	0.553
Prothrombin time (INR)	1.4 (1.2–1.7)	1.4 (1.2–1.7)	1.4 (1.2–1.6)	0.923
Total bilirubin (mg/dL)	1.9 (1.1–4.5)	1.9 (1.1–4.5)	1.9 (1.2–4.2)	0.717
Creatinine (mg/dL)	0.8 ± 0.2	0.8 ± 0.2	0.8 ± 0.2	0.974
Brain natriuretic peptide (pg/mL)	40.0 (19.0–84.0)	42.0 (20.0–85.0)	34.0 (16.0–83.0)	0.005
**Donor variables**				
Donor age (years)	26.0 (21.0–32.0)	26.0 (21.0–32.0)	27.0 (23.0–32.0)	0.001
Donor sex, male	1319 (72.0%)	1021 (73.4%)	298 (67.4%)	0.017
Donor body mass index (kg/m^2^)	22.9 ± 2.9	22.9 ± 2.9	23.1 ± 2.9	0.201
Total fatty change (%)	1.0 (0.0–5.0)	1.0 (0.0–5.0)	2.0 (0.0–5.0)	0.199
Macro fatty change (%)	1.0 (0.0–3.0)	1.0 (0.0–3.0)	1.0 (0.0–3.0)	0.295
**Intraoperative data**				
Graft-to-recipient weight ratio	1.1 ± 0.3	1.1 ± 0.2	1.2 ± 0.3	0.003
Postreperfusion syndrome	714 (39.0%)	542 (39.0%)	172 (38.9%)	1.000
Cold ischaemic time (min)	81.0 (66.0–98.0)	81.0 (67.0–98.0)	82.0 (65.0–100.0)	0.872
Warm ischaemic time (min)	40.0 (33.0–50.0)	40.0 (32.0–48.0)	43.0 (35.0–57.0)	<0.001
Operation time (min)	840.0 (760.0–930.0)	822.0 (750.0–905.0)	886.5 (805.0–975.0)	<0.001
**Fluid balance**				
Crystalloid (L)	6.3 (5.0–7.9)	6.0 (4.8–7.2)	7.7 (6.0–10.3)	<0.001
Albumin (L)	3.0 (2.3–4.0)	3.0 (2.0–3.8)	3.3 (2.5–5.0)	<0.001
Packed red blood cell transfusion (units)	6.0 (2.0–12.0)	6.0 (2.0–11.0)	9.0 (4.0–18.0)	<0.001
Fresh frozen plasma transfusion (units)	7.0 (2.0–14.0)	6.0 (2.0–12.0)	10.0 (5.0–20.0)	<0.001
Urine output (L)	1.5 (1.0–2.0)	1.4 (1.0–1.9)	1.6 (1.2–2.4)	<0.001
Epinephrine use	687 (37.5%)	521 (37.5%)	166 (37.6%)	1.000
Vasopressor use	1083 (59.1%)	848 (61.0%)	235 (53.2%)	0.004

INR, international normalized ratio.

### Graft failure

**Figure 2:**
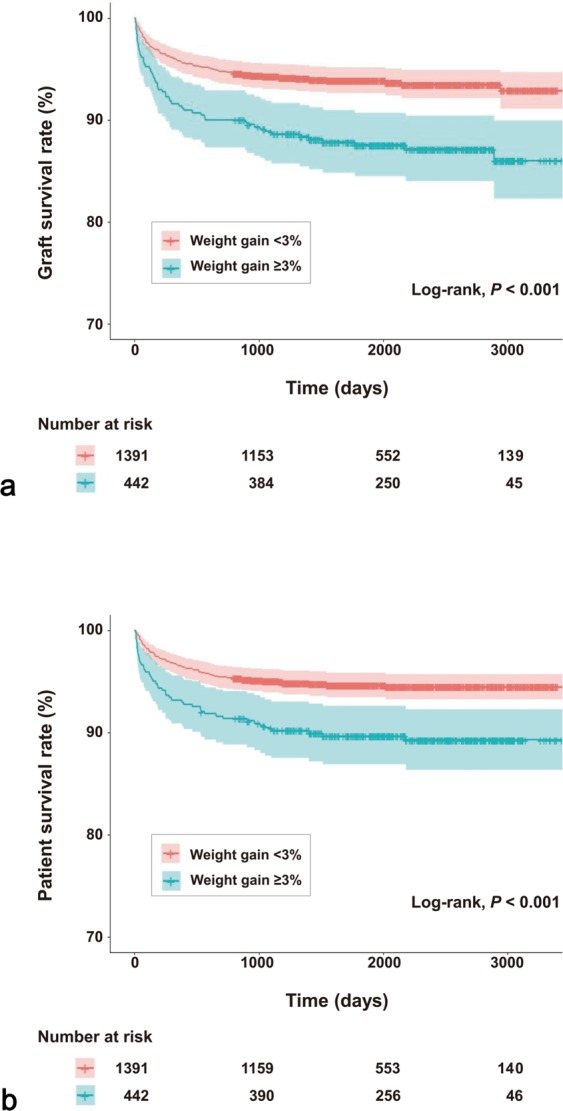
(**a**) Graft and (**b**) patient survival rates stratified by postoperative weight gain (<3% or ≥3%) in living donor liver transplantation recipients. Survival curves are provided with 95% confidence intervals.

The incidence of graft failure was 6.2% (n = 86) in the <3% group and 12.7% (n = 56) in the ≥3% group (*P* < 0.001). Postoperative weight gain ≥3% was associated with a significantly higher rate of graft failure (HR, 2.047; 95% CI, 1.462–2.867; *P* < 0.001) in the univariate Cox proportional hazards model. Other variables related to graft failure are presented in Table 2. After adjusting for multiple confounders, postoperative weight gain ≥3% was still associated with a higher incidence of graft failure (aHR, 1.763; 95% CI, 1.248–2.490; *P* = 0.001). Kaplan-Meier survival analysis revealed that the ≥3% group had a significantly higher incidence of graft failure than the <3% group (log-rank test, *P* < 0.001) (Fig. 2a).

**Table 2:** Cox proportional hazards regression analysis of factors associated with graft failure in living donor liver transplantation recipients.

	Unadjusted				Multivariable adjusted^a^			
	HR	95% CI		*P*-value	HR	95% CI		*P*-value
Age (years)	1.034	1.011	1.058	0.003	1.028	1.006	1.050	0.013
Sex, male	0.641	0.452	0.909	0.013				
Body mass index (kg/m^2^)	1.008	0.958	1.061	0.752				
Diabetes mellitus	1.325	0.911	1.927	0.142				
Hypertension	0.718	0.414	1.247	0.239				
Coronary arterial disease	0.591	0.188	1.854	0.367				
Fulminant hepatic failure	2.242	1.241	4.050	0.007	2.526	1.344	4.744	0.004
Model for end-stage liver disease score	1.037	1.017	1.057	<0.001				
Hepatic encephalopathy	1.844	1.238	2.746	0.003				
Ascites	1.397	0.994	1.963	0.054				
Spontaneous bacterial peritonitis	1.343	0.706	2.554	0.369				
Combined hepatocellular carcinoma	0.931	0.670	1.293	0.669				
Brain natriuretic peptide (pg/mL)	1.001	1.001	1.002	<0.001	1.001	1.001	1.002	<0.001
Donor age (years)	1.029	1.011	1.049	0.002	1.021	1.001	1.042	0.041
Donor sex, male	0.997	0.692	1.438	0.989				
Donor body mass index (kg/m^2^)	1.042	0.984	1.102	0.156				
Total fatty change (%)	1.019	0.995	1.043	0.119				
Macro fatty change (%)	1.024	0.990	1.059	0.172				
Graft-to-recipient weight ratio	1.650	0.884	3.079	0.116				
Postreperfusion syndrome	1.587	1.142	2.206	0.006				
Cold ischaemic time (min)	1.000	0.997	1.003	0.973				
Warm ischaemic time (min)	1.001	0.997	1.005	0.547				
Operation time (h)	1.082	1.014	1.154	0.017				
Packed red blood cell transfusion (units)	1.019	1.012	1.026	<0.001	1.015	1.007	1.023	<0.001
Epinephrine use	1.585	1.141	2.204	0.006				
Vasopressor use	1.088	0.777	1.523	0.623				
Postoperative weight gain ≥3%	2.047	1.462	2.867	<0.001	1.763	1.248	2.490	0.001

HR, hazard ratio; CI, confidence interval. ^a^Adjusted for all variables in the table.

**Figure 3:**
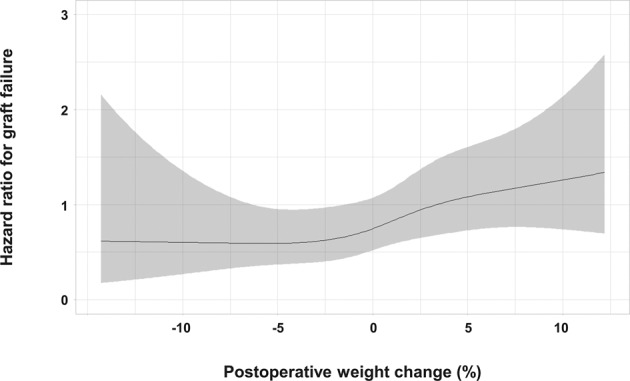
Postoperative weight change and risk of graft failure in living donor liver transplantation recipients. The solid black line represents the adjusted hazard ratio for the association between postoperative weight change and graft failure, and grey shading represents the 95% confidence interval of the estimate. Postoperative weight change was modelled with restricted cubic splines with 5 knots (5^th^, 27.5^th^, 50^th^, 72.5^th^, and 95^th^ percentiles) in a multivariate Cox model, which was adjusted for all variables in Table 2.

In addition, we analysed the nonlinear relationship between postoperative weight change (%) and the risk of graft failure by entering postoperative weight change modelled with restricted cubic splines in a multivariate Cox regression model. This model was adjusted for all variables in Table 2. Variables with *P*-values < 0.1 in univariate Cox analysis were selected and included in the final model, which is shown in Fig. 3. After adjusting for confounding factors, LT recipients with postoperative weight gain had higher likelihood of graft failure than those with postoperative weight loss. In addition, postoperative weight change (%) was entered as a continuous variable in a multivariate Cox model for graft failure, which was adjusted for all variables in Table 2. The aHR for each 1% increase in postoperative weight was 1.045 (95% CI, 1.009–1.082; *P* = 0.015).

### Other adverse outcomes

Multiple logistic regression analysis revealed that postoperative weight gain ≥3% was associated with EAD (aOR, 1.553; 95% CI, 1.024–2.356; *P* = 0.038) (Table 3). Multivariate Cox proportional regression analysis revealed that postoperative weight gain ≥3% was associated with overall mortality (aHR, 1.731; 95% CI, 1.182–2.535; *P* = 0.005). Kaplan-Meier survival analysis revealed that the ≥3% group had a significantly higher overall mortality rate than the <3% group (log-rank test, *P* < 0.001) (Fig. 2b). The ≥3% group had significantly longer ICU stay (median 3.0 days [IQR, 2.0–6.0] versus 3.0 days [2.0–5.0]; *P* < 0.001) and hospital stay (34.0 days [27.0–51.0] versus 31.0 days [25.0–43.0]; *P* < 0.001) than the <3% group. In addition, we tested our hypothesis with postoperative weight gain cut-off value of 5% (Supplementary Tables S1, S2 and Fig. S1).

**Table 3:** Predictive value of postoperative weight gain ≥3% for clinical outcomes in living donor liver transplantation recipients.

		Event/n	Unadjusted				Multivariable adjusted^a^			
			OR	95% CI		*P*-value	OR	95% CI		*P*-value
Early allograft dysfunction	Weight gain ≥3% Weight gain <3%	60/442 130/1391	1.524 1	1.099	2.112	0.012	1.553 1	1.024	2.356	0.038
		**Event/n**	**HR**	**95% CI**		***P*-value**	**HR**	**95% CI**		***P*-value**
Graft failure	Weight gain ≥3% Weight gain <3%	56/442 86/1391	2.047 1	1.462	2.867	<0.001	1.763 1	1.248	2.490	0.001
Overall mortality	Weight gain ≥3% Weight gain <3%	46/442 73/1391	1.990 1	1.376	2.879	<0.001	1.731 1	1.182	2.535	0.005

OR, odds ratio; HR, hazard ratio; CI, confidence interval. ^a^Adjusted for all variables in Table 2.

There was no difference in the pulmonary complication rates such as pulmonary edema (222 [16.0%] vs. 66 [14.9%], *P* = 0.658), pneumonia (94 [6.8%] vs. 23 [5.2%], *P* = 0.292), and acute respiratory distress syndrome (1 [0.1%] vs. 2 [0.5%], *P* = 0.294) between the postoperative weight gain <3% and ≥3% groups. In addition, no difference was found in the incidence of AKI according to the KDIGO criteria (990 [71.3%] vs. 314 [71.0%], *P* = 0.956) between the two groups.

### Postoperative inflammatory markers

**Figure 4:**
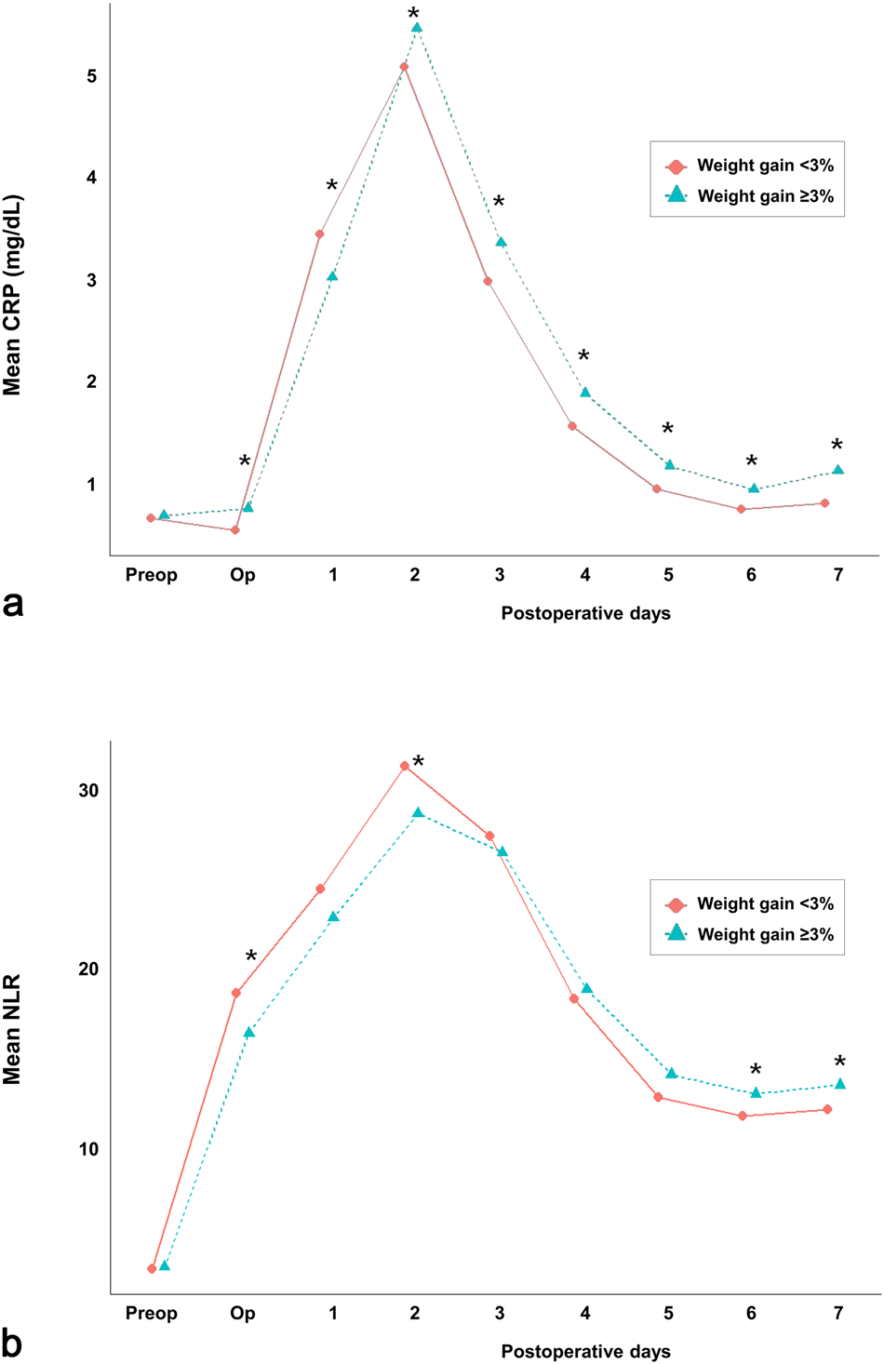
Sequential changes in mean (**a**) CRP and (**b**) NLR values in living donor liver transplantation recipients, *P < 0.05. Abbreviations: CRP, C-reactive protein; NLR, neutrophil-to-lymphocyte ratio; Preop, preoperative day; Op, operation day.

The mean CRP and NLR levels are presented consecutively from 1 day before surgery to POD 7 (Fig. 4). The mean CRP levels were significantly higher in the ≥3% group than in the <3% group from POD 2 onwards (Fig. 4a). The mean NLR values were higher in the ≥3% group than in the <3% group starting from POD 4, and statistical significance was achieved from POD 6 onwards (Fig. 4b).

## Discussion

In this retrospective study of 1833 LDLT recipients, we found that early postoperative weight gain was associated with adverse postoperative outcomes. Higher postoperative weight gain was associated with inferior graft survival in LDLT recipients even after adjusting for previously identified risk factors. In addition, recipients with postoperative weight gain ≥3% (as opposed to weight gain <3%) had significantly higher risks of graft failure, EAD, and mortality before and after multivariable adjustment.

Although fluid therapy plays an important role in perioperative care, the optimal approach for fluid administration in patients undergoing major surgery is still controversial^29–32^. Dehydration can lead to dysfunction of vital organs, and adequate fluid administration is essential for maintaining optimal cardiac output, tissue perfusion, and oxygen delivery^33,34^. However, FO has also been reported to be detrimental by affecting multiple organ systems^13,14^, resulting in postoperative complications, prolonged hospital stay, and increased mortality in critically ill patients or those undergoing major abdominal surgery^1–6^. Our findings suggest that even a mild degree of FO may be harmful for graft or patient survival in LDLT, making it prudent to avoid postoperative weight gain ≥3% in these patients.

In LT recipients, only a few studies have addressed the association between fluid administration and graft or patient survival^8,9^. In a previous study, the total volume of intraoperative fluid administration was found to be associated with adverse outcomes (either in-hospital death or prolonged postoperative hospitalization [>14 days] associated with morbidity) in LT recipients^8^. After adjusting for age, liver disease severity, intraoperative transfusion volume, inotropic use, and operation time, the volume of intraoperative fluid administration was still independently associated with adverse outcomes. In another study of 1197 LT recipients, a negative correlation was observed between ICU readmission and graft or patient survival^9^. In the subgroup analysis of 53 LT recipients in that study, intravascular FO was suspected to be a significant factor for decompensation leading to ICU readmission in the recipients. Our study is in line with the above studies that addressed the deleterious effects of FO on post-LT outcomes. Moreover, our findings further identified the direct relevance of FO to post-LT graft failure in LDLT recipients.

FO may be causally linked to the development of graft failure in LT recipients. Perioperative acute hypervolaemia may destroy the endothelial surface layer along with the inevitable damage caused by surgical trauma (such as mechanical stress and ischaemia-reperfusion injury), leading to pathologic shifts of fluid and protein towards the interstitium^15^. This is reflected by postoperative weight gain, an indirect measure for the interstitial fluid shift, which leads to bacterial translocation, elevated endotoxin levels, and systemic inflammation^15,16^. Furthermore, pathologic inflammation and endothelial dysfunction are known to promote angiogenesis, fibrogenesis, cirrhosis, and increased hepatic resistance, ultimately resulting in portal hypertension and decreased effective hepatocyte perfusion with the risk of liver failure^18–20,35^. Specifically, CRP has been reported to be associated with extracellular volume expansion^36^ and to have a direct role in endothelial activation and atherosclerosis formation^37,38^. Elevated levels of CRP (preoperatively, early postoperatively, or >1 year after transplantation) have been found to be associated with inferior graft survival rates; increased intimal thickening and stenosis after arterial allograft transplantation^39^, higher risk of EAD and mortality after LT^40^, higher risk of coronary artery disease progression and graft failure after heart transplantation^38^, and rapid deterioration of graft function after renal transplantation^41^.

In this study, in agreement with the above studies that elaborated the association between FO, inflammation, and organ or graft dysfunction^15,16,18–20,35–41^, the postoperative weight gain ≥3% group had significantly greater mean CRP levels from POD 2 and significantly higher risks of EAD, graft failure, and mortality than the postoperative weight gain <3% group. Previous studies have reported that patients who gain at least 2.5–3 kg of body weight after major abdominal surgery have poorer clinical outcomes than those who maintained a state of zero fluid balance^1–3,31^. Our findings also suggest that even a mild postoperative weight gain could be associated with inferior graft and patient survival after LT. However, considering the potential harmful effects of volume depletion on other organs, a negative fluid-balance might also have adverse effects in LT recipients. Therefore, we presume that careful fluid administration (in terms of type, volume, and timing) would be necessary in LDLT recipients for maintaining normovolaemia, to ensure adequate perfusion of multiple organs and at the same time to avoid postoperative weight gain^15,42^.

Patient or graft survival after LT has been reported to be inversely related to older donor age, older recipient age, acute liver failure, and higher RBC transfusion requirements^11,12,43,44^. Preoperative BNP level has also been found to be an independent predictor of post-LT ICU mortality^45^. Therefore, our findings of the factors associated with graft failure are in line with previous findings.

This study has some limitations. Because of the retrospective design, the effects of confounding factors on post-LT graft failure could not be completely ruled out. Further, although a causal relationship between postoperative weight gain and graft failure could be inferred based on the physiology or previous studies, it could not be confirmed. Additionally, because the data were collected from a single centre, idiosyncrasies of local surgical and perioperative management of patients may have affected clinical outcomes. Therefore, large-scale multicentre prospective studies are needed to confirm the causal relationship between postoperative weight gain and graft failure in LDLT recipients.

In conclusion, the results of this large observational study suggest that early postoperative weight gain may be associated with systemic inflammation and deterioration of graft function in LDLT recipients. Additionally, postoperative weight gain ≥3% (as opposed to weight gain <3%) was associated with significantly higher risks of EAD and mortality after LDLT.

## Supplementary information

Supplementary information 

## Data Availability

The datasets generated during and/or analysed during the current study are available from the corresponding author on reasonable request.
