# Supplementary material for: Early postoperative weight gain is associated with increased risk of graft failure in living donor liver transplant recipients
Source: Sci Rep. 2019 Dec 27;9:20096. doi: 10.1038/s41598-019-56543-3 (PMC6934543; doi:10.1038/s41598-019-56543-3)

Supplementary Materials

Early postoperative weight gain is associated with increased risk of graft failure in living donor liver transplant recipients

Hye-Won Jeong, Kyeo-Woon Jung, Seon-Ok Kim, Hye-Mee Kwon, Young-Jin Moon, In-Gu Jun, Jun-Gol Song, Gyu-Sam Hwang

Contents

Supplementary Table S1. Cox proportional hazards regression analysis of factors associated with graft failure in living donor liver transplantation recipients

Supplementary Table S2. Predictive value of postoperative weight gain ≥5% for clinical outcomes in living donor liver transplantation recipients

Supplementary Figure S1. Graft and patient survival rates stratified by postoperative weight gain (<5% or ≥5%) in living donor liver transplantation recipients

Supplementary Table S1. Cox proportional hazards regression analysis of factors associated with graft failure in living donor liver transplantation recipients. HR, hazard ratio; CI, confidence interval; [a]Adjusted for all variables in the table.

	Unadjusted				Multivariable adjusted[a]			
	**HR**	**95% CI**		***P*-value**	**HR**	**95% CI**		***P*-value**
Age (years)	1.034	1.011	1.058	0.003	1.028	1.006	1.050	0.013
Sex, male	0.641	0.452	0.909	0.013				
Body mass index (kg/m^2)	1.008	0.958	1.061	0.752				
Diabetes mellitus	1.325	0.911	1.927	0.142				
Hypertension	0.718	0.414	1.247	0.239				
Coronary arterial disease	0.591	0.188	1.854	0.367				
Fulminant hepatic failure	2.242	1.241	4.050	0.007	2.364	1.259	4.438	0.007
Model for end-stage liver disease score	1.037	1.017	1.057	< 0.001				
Hepatic encephalopathy	1.844	1.238	2.746	0.003				
Ascites	1.397	0.994	1.963	0.054				
Spontaneous bacterial peritonitis	1.343	0.706	2.554	0.369				
Combined hepatocellular carcinoma	0.931	0.670	1.293	0.669				
Brain natriuretic peptide (pg/mL)	1.001	1.001	1.002	< 0.001	1.001	1.000	1.002	0.001
Donor age (years)	1.029	1.011	1.049	0.002	1.020	1.000	1.041	0.049
Donor sex, male	0.997	0.692	1.438	0.989				
Donor body mass index (kg/m^2)	1.042	0.984	1.102	0.156				
Total fatty change (%)	1.019	0.995	1.043	0.119				
Macro fatty change (%)	1.024	0.990	1.059	0.172				
Graft-to-recipient weight ratio	1.650	0.884	3.079	0.116				
Postreperfusion syndrome	1.587	1.142	2.206	0.006				
Cold ischaemic time (min)	1.000	0.997	1.003	0.973				
Warm ischaemic time (min)	1.001	0.997	1.005	0.547				
Operation time (h)	1.082	1.014	1.154	0.017				
Packed red blood cell transfusion (units)	1.019	1.012	1.026	< 0.001	1.015	1.007	1.023	< 0.001
Epinephrine use	1.585	1.141	2.204	0.006				
Vasopressor use	1.088	0.777	1.523	0.623				
Postoperative weight gain ≥5%	2.241	1.511	3.322	< 0.001	1.645	1.087	2.489	0.018

Supplementary Table S2. Predictive value of postoperative weight gain ≥5% for clinical outcomes in living donor liver transplantation recipients. OR, odds ratio; HR, hazard ratio; CI, confidence interval; [a]Adjusted for all variables in Supplementary Table S1.

			Unadjusted				Multivariable adjusted[a]			
		**Event/n**	**OR**	**95% CI**		***P*-value**	**OR**	**95% CI**		***P*-value**
Early allograft dysfunction	Weight gain ≥5%	48/212	3.048	2.117	4.391	< 0.001	2.195	1.371	3.515	0.001
	Weight gain <5%	142/1621	1				1			
		**Event/n**	**HR**	**95% CI**		***P*-value**	**HR**	**95% CI**		***P*-value**
Graft failure	Weight gain ≥5%	32/212	2.240	1.511	3.322	< 0.001	1.645	1.087	2.489	0.018
	Weight gain <5%	110/1621	1				1			
Overall mortality	Weight gain ≥5%	26/212	2.166	1.402	3.346	< 0.001	1.615	1.023	2.551	0.040
	Weight gain <5%	93/1621	1				1			

Supplementary Fig. S1. (a) Graft and (b) patient survival rates stratified by postoperative weight gain (<5% or ≥5%) in living donor liver transplantation recipients. Survival curves are provided with 95% confidence intervals.

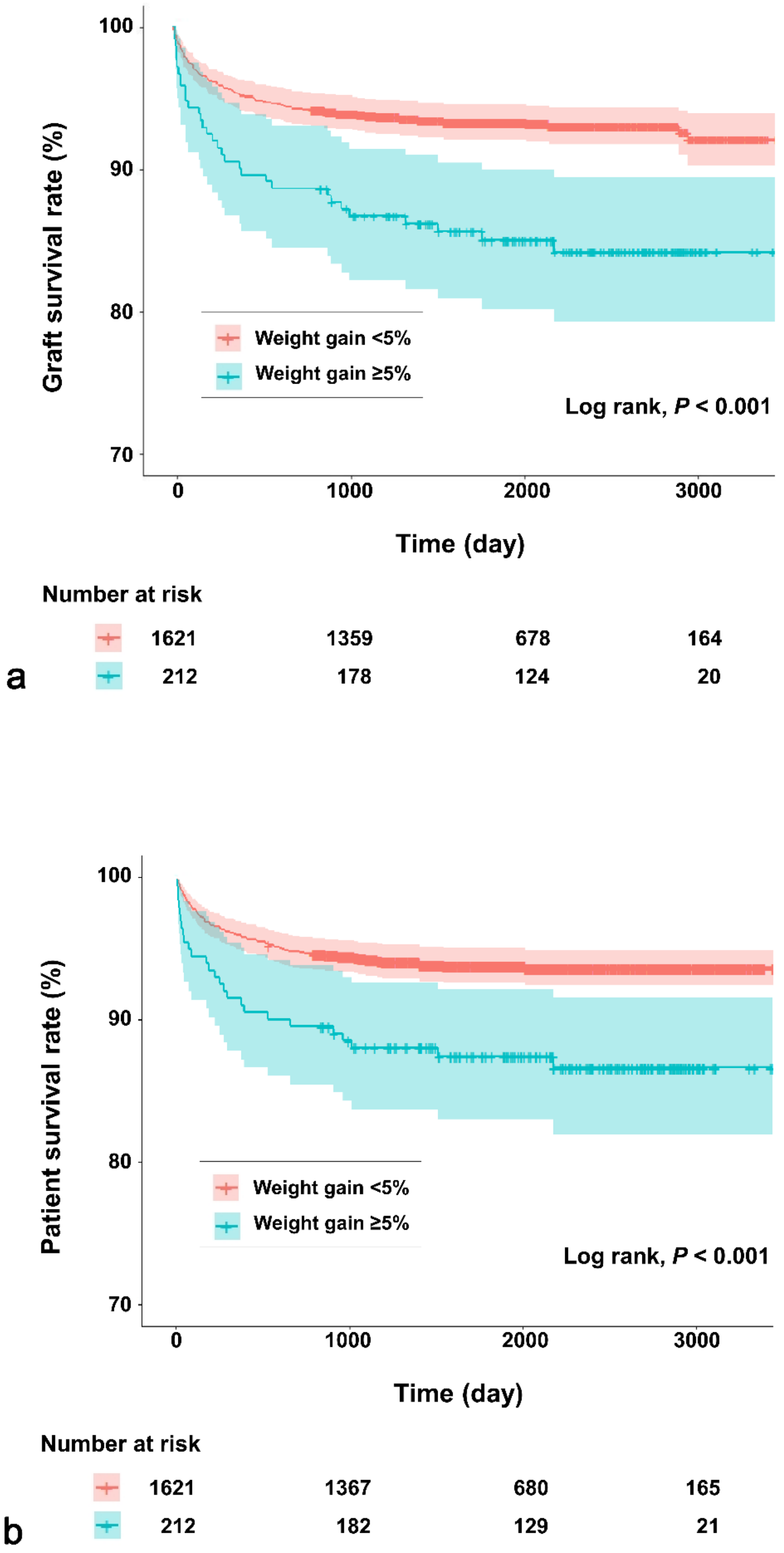

Supplement: Supplementary file 1 — Supplementary information [file 41598_2019_56543_MOESM1_ESM.pdf]
